# Flood‐Driven Microbial Resurgence: Functional Shifts and Beneficial Taxa in Post‐Flood Agricultural and Residential Soils in Bangladesh

**DOI:** 10.1002/mbo3.70069

**Published:** 2025-10-14

**Authors:** Tanzim Rahman, S. M. Arefeen Haider, Fatema Tuz Zohra Khan, Md. Shafiqul Islam, Zahid Hayat Mahmud, Muhammad Manjurul Karim, Mustafizur Rahman, Mohammad Jubair

**Affiliations:** ^1^ Laboratory of Environmental Health, Health Systems and Population Studies Division, International Centre for Diarrhoeal Disease Research, Bangladesh (icddr,b) Dhaka Bangladesh; ^2^ Genome Centre, Infectious Diseases Division, International Centre for Diarrhoeal Disease Research, Bangladesh (icddr,b) Dhaka Bangladesh; ^3^ Department of Microbiology University of Dhaka Dhaka 1000 Bangladesh

**Keywords:** 2024 floods, Bangladesh, flooding impacts, microbial diversity, soil beneficial microbes

## Abstract

Flooding events alter microbial communities, impacting soil health and ecosystem recovery. This study examined the 2024 Bangladesh floods' effects on microbial diversity in agricultural and residential soils. We collected paired soil samples from six flooded and two non‐flooded sites within a single rural subdistrict after the floods. This sample size was logistically feasible for an initial survey and provides a foundational data set for future larger‐scale investigations. Our findings revealed that flooding increased microbial diversity and facilitated the dispersal of unique taxa, with 45 species shared between flooded groups compared to only 6 in non‐flooded controls. Notably, 29 beneficial microbes introduced post‐flood were identified, categorized into 8 functional groups critical for soil recovery. Residential soils were enriched in nitrogen‐fixing microbes, whereas agricultural soils showed higher abundance of phosphate solubilizers and plant growth‐promoting rhizobacteria. Sulfur/iron cyclers and cyanobacteria demonstrated flood‐adaptive roles. Pathogen screening identified no canonical human pathogens with the exception of *Clostridium disporicum*, detected only in one non‐flooded sample. Archaea from *Woesearchaeales* and bacteria from the *Gemmataceae* dominated the microbial communities. Flooded environments harbored unique taxa, such as *Sulfuricurvum* sp. and the *Nitrospira* genus, which were absent in controls. Alpha diversity analysis revealed a higher Chao1 richness in flooded soils compared to controls, although statistical significance was not found due to insufficient sample size. Beta diversity showed greater variability between flooded soils relative to non‐flooded controls, also not statistically significant. These findings demonstrate that flooding can act as a driver for dispersing beneficial microbes, supporting soil restoration and enhancing agricultural resilience.

## Graphical abstract

Flooding in Bangladesh increased microbial diversity and introduced 29 beneficial taxa, including nitrogen fixers and phosphate solubilizers, enhancing soil recovery. Unique flood‐adapted microbes like *Sulfuricurvum* sp. emerged, highlighting flooding's role in reshaping microbial communities for agricultural resilience.

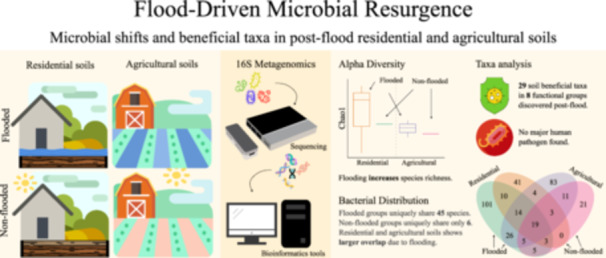

## 1 Introduction

Flooding is a natural disturbance that significantly alters soil ecosystems by reshaping microbial communities and generating new ecological niches shaped by changing physicochemical conditions. Although often associated with disruption and damage, flooding can also have beneficial effects on soil microbiomes, enhancing microbial diversity, nutrient cycling, and ecosystem functioning (Fierer et al. 2013). Inundation facilitates the dispersal of microorganisms, leading to increased species overlap between previously distinct habitats and fostering the emergence of unique microbial assemblages (Baldwin et al. 2020). Such changes can enhance soil health, especially in agricultural systems, where microbial processes drive nutrient cycling, organic matter breakdown, and plant productivity (Tiedje et al. 2022). Understanding how flooding influences microbial communities is essential for developing strategies to enhance soil fertility and ecosystem resilience in flood‐prone regions.

In Bangladesh, the connection between flooding and soil fertility is deeply rooted in the country's agricultural traditions. For centuries, farmers have relied on seasonal floods to replenish soil nutrients, particularly in the deltaic regions where silt deposition from riverine flooding enriches the soil with organic matter and vital minerals (Brammer 2014). This natural process, often referred to as “floodplain agriculture,” has maintained crop productivity and supported livelihoods in a region where farming is the backbone of the economy. However, the benefits of flooding extend beyond nutrient deposition; they also include the boosting of microbial activity, which plays a pivotal role in nutrient cycling and soil health (Islam et al. 2021).

Bangladesh, situated on the world's largest delta, is highly susceptible to flooding due to its extensive river network, low‐lying topography, and tropical monsoon climate. Heavy rainfall during the monsoon season (June to October) frequently leads to widespread flooding, particularly in low‐lying regions (Li et al. 2023). These floods, while often devastating, provide a unique opportunity to study how microbial communities adapt and thrive under dynamic environmental conditions. The interplay between flooding and soil microbiomes is particularly relevant in Bangladesh, where agriculture is a cornerstone of the economy and livelihoods for millions of people (Rahman et al. 2022).

The traditional practice of floodplain agriculture in Bangladesh highlights the dual role of flooding: while it can cause immediate disruption, it also fosters long‐term soil fertility through nutrient deposition and microbial activity. Flooding introduces organic matter and nutrients into the soil, which serve as substrates for microbial growth and metabolism (Brammer 2014). Additionally, the anaerobic conditions created by flooding can stimulate the activity of specific microbial groups, such as methanogens, which produce methane, and sulfate‐reducing bacteria, which play key roles in anaerobic decomposition and sulfur cycling, which contribute to nutrient cycling and organic matter decomposition (Tiedje et al. 2022). These microbial shifts can enhance soil fertility and support crop productivity in the post‐flood period.

In August 2024, Bangladesh experienced one of the most severe flooding events in recent history, particularly affecting the eastern regions, including Feni, Noakhali, Lakshmipur, and Comilla. The floods were triggered by abnormally heavy rainfall and the release of water from dams in neighboring India (The Daily Star 2024). Among these regions, Feni district was the most severely impacted, with widespread property damage, crop loss, and mass displacement (Alam et al. 2024). The scale of the flooding provided a unique opportunity to investigate the effects of inundation on soil microbiomes in one of the hardest‐hit areas. Our study focused on Chhagalnaiya subdistrict in Feni—a representative example of Bangladesh's climatic vulnerability and dependence on agriculture—to examine how flooding alters soil microbiomes (Hossain et al. 2023).

Using full‐length 16S rRNA sequencing on the Oxford Nanopore platform, this study characterizes microbial diversity in agricultural and residential soils from both flooded and non‐flooded areas following the 2024 monsoon floods (Bolyen et al. 2019). We hypothesized that flooding enriches soil microbiomes with functionally diverse taxa, enhancing nutrient cycling and ecosystem recovery. These shifts may highlight the potential for flooding to enrich soil microbiomes and improve ecosystem functioning, even in the face of environmental disruption.

By examining the microbial dynamics in flood‐affected soils, this study provides critical insights into the ecological impacts of flooding and offers a foundation for targeted strategies to enhance soil health and resilience in flood‐prone regions. Future research should focus on longitudinal sampling, expanded geographic coverage, and functional metagenomics to further explore the beneficial effects of flooding on microbial communities and their roles in ecosystem stability. This study not only advances our understanding of flood‐induced microbial shifts but also contributes to the development of sustainable agricultural practices in vulnerable regions like Bangladesh.

## 2 Methods

### 2.1 Sample Collection and Preparation

**Figure 1:**
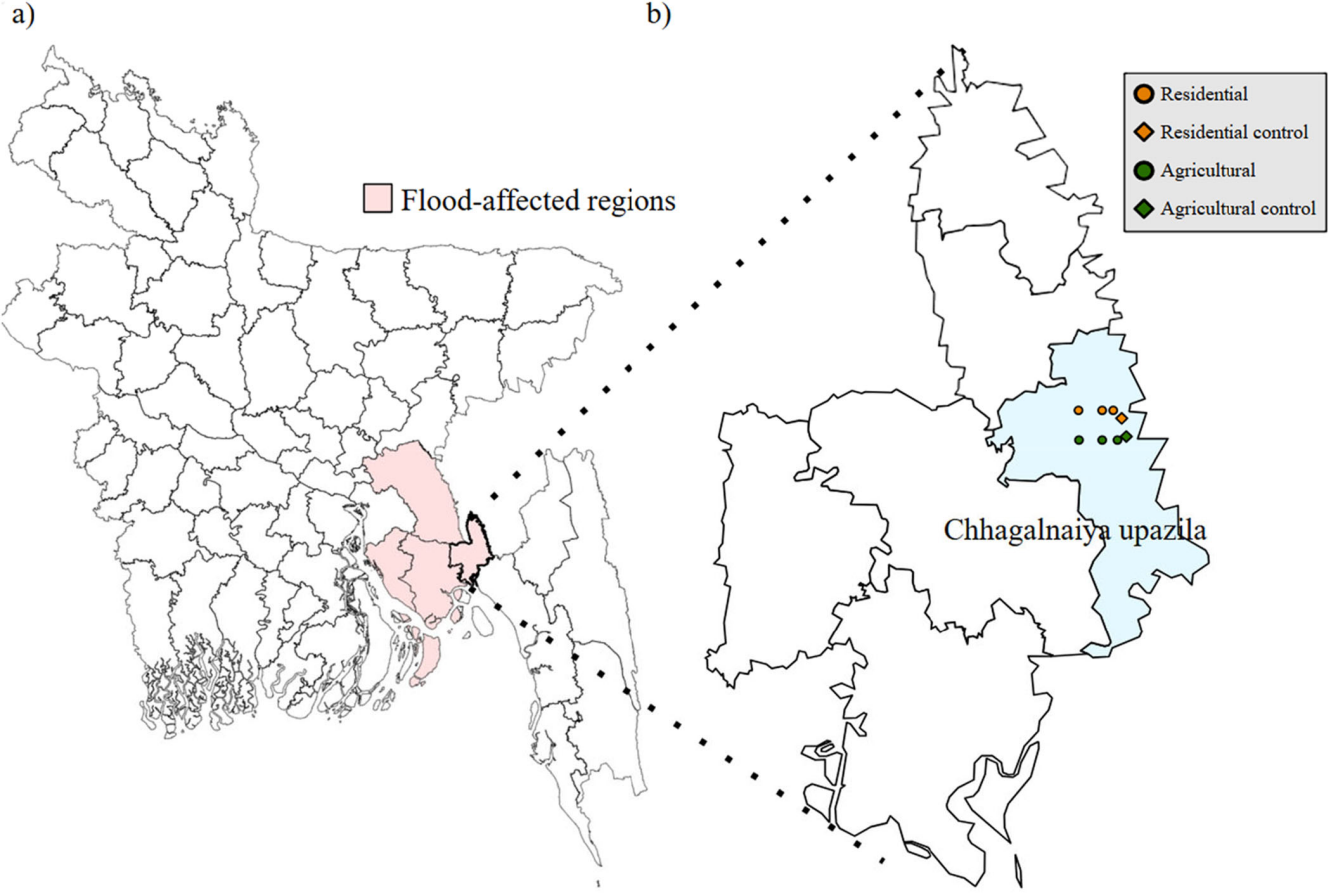
(a) Map of Bangladesh showing flood‐affected regions during the 2024 floods and (b) a zoomed‐in view of Feni district, highlighting the Chhagalnaiya upazila as well as the eight sample sites.

A total of eight samples were collected from the top 15 cm of the soil profile at regions near Chhagalnaiya, Feni, in September 2024 to analyze the impacts of flooding. The sample size (*n* = 8) was determined based on a paired, comparative study design focused on feasibility and logistical constraints following a major flood event. This approach prioritized controlled comparisons over large‐scale surveying. Figure 1 illustrates the sampling sites, with labels specifying the type of sample collected. Three residential samples were obtained from soil within residential areas in flood‐affected zones, while three agricultural samples were collected from soil in flooded agricultural fields. To facilitate comparative analysis, a residential control sample and an agricultural control sample were also collected from non‐flooded areas. All samples were collected using a sterile stainless‐steel soil auger, placed in sealed, sterile Whirl‐Pak® bags, and stored on ice in a cooler for transport. Within 6 h of collection, samples were transferred to a −20°C freezer for preservation until DNA extraction and chemical analysis.

### 2.2 DNA Extraction

DNA was extracted from all soil samples using the DNeasy PowerSoil Pro Kit (Qiagen, 47014). Following the manufacturer's protocol, 500 mg of soil was processed for DNA isolation. The purity and concentration of the extracted DNA were evaluated using a Nanodrop spectrophotometer and a Qubit 4.0 Fluorometer, respectively. High‐quality DNA was confirmed by a 260/280 ratio of ~1.8 and a 260/230 ratio of ~2.0.

### 2.3 Library Preparation

For library preparation, the 16S Barcoding Kit 24 V14 (SQK‐16S114.24) was used. Genomic DNA samples were prepared by diluting 10 ng of DNA to a final volume of 15 μL with nuclease‐free water. A PCR mixture was prepared using LongAmp Hot Start Taq 2X Master Mix, and barcodes were added for sample identification. Amplification was performed under specific cycling conditions, followed by the addition of EDTA to stop the reaction. Barcoded samples were pooled in equimolar ratios, purified using AMPure XP Beads, and quantified. A diluted Rapid Adapter mixture was added to the barcoded DNA, and the prepared libraries were sequenced using the MinION platform.

### 2.4 Bioinformatics Workflow

Full‐length 16S rRNA amplicons were generated using Oxford Nanopore Technologies (ONT). Subsequent bioinformatics analysis on the microbiome was performed using QIIME 2 version 2020.8.0 (Bolyen et al. 2019). Raw single‐end sequence data was imported into QIIME 2 and processed as a QIIME artifact. The q2‐vsearch plugin (Rognes et al. 2016) was utilized for dereplication, chimera removal, and clustering. Dereplication consolidated identical sequences into unique features, producing a feature table and representative sequences. Chimeric sequences were identified and filtered by aligning against the SILVA 138 reference database (Quast et al. 2012; Yilmaz et al. 2013). Open‐reference clustering (Rideout et al. 2014) was then performed on the non‐chimeric sequences using the same database, clustering features into Operational Taxonomic Units (OTUs) at an 85% sequence similarity threshold. A Naive Bayes classifier was trained using RESCRIPt (Robeson et al. 2021), using the Silva 138 SSURef NR99 full‐length sequences. Taxonomy was assigned to the non‐chimeric sequences using this classifier via the q2‐feature‐classifier plugin (Bokulich et al. 2018). Finally, interactive visualizations were generated using the tabulate method from the q2‐metadata plugin, enabling detailed exploration of the results.

### 2.5 Statistical Analysis

A custom Python script was used to convert the taxonomic table generated at the end of the bioinformatics workflow into a Phyloseq object (McMurdle and Holmes 2013). Subsequent statistical analyses were performed using custom R scripts leveraging the *microeco* package (Liu et al. 2020). To assess the diversity and composition of microbial communities in flooded and non‐flooded environments, both alpha and beta diversity analyses were conducted. Alpha diversity, which measures the diversity within individual samples, was assessed using the Chao1 diversity index, providing insights into the species richness within each sample group. Beta diversity, which evaluates the differences in microbial community composition between samples, was analyzed using Bray‐Curtis dissimilarity, comparing both within‐group and between‐group samples. Additionally, principal coordinate analysis (PCoA) was performed based on Bray‐Curtis dissimilarity to visualize community differences. Finally, the Wilcoxon Rank Sum test was applied to the alpha and beta diversity results to evaluate statistical significance. Given the limited sample size, which was insufficient for achieving statistical power, the statistical analysis was intentionally exploratory rather than confirmatory.

### 2.6 Soil Beneficial Microbes Screening

Microbes introduced after flooding in agricultural and residential soils were isolated by excluding those from residential and agricultural control samples to focus solely on post‐flood introductions. The refined list was cross‐referenced with established databases (e.g., NCBI Taxonomy, BRENDA) and peer‐reviewed literature (e.g., Bergey's Manual, Fierer et al. 2007) to confirm their roles as beneficial soil microbes. Functional categories (e.g., nitrogen fixation, organic decomposition) and soil locations (agricultural, residential, or both) were systematically assigned, ensuring alignment with validated references. Duplicates and microbes with ambiguous roles were removed to maintain clarity and accuracy.

### 2.7 Pathogen Screening

To identify and filter out potential human pathogenic species, a pathogenic screening process was carried out on the resulting taxonomy profile. First, a comprehensive list of known pathogens was downloaded from the NCBI pathogen database (NCBI Pathogen Database 2024). This list was then cross‐referenced with the taxonomic classifications obtained from the metagenomics analysis. By comparing the two datasets, known pathogens present in the soil samples could be identified.

### 2.8 Quality Assurance

To confirm the proper collection and processing of soil samples in this study, a quality assurance step was implemented by comparing the taxonomic profiles of these samples with those from an external project. The external project, conducted in Khulna, involved both aerobic and anaerobic soil samples [REF: https://www.ncbi.nlm.nih.gov/bioproject/PRJNA1123412]. From this data set, eight aerobic soil samples were randomly selected for comparison, as their environmental conditions more closely resembled those of the samples in this study. The geographic proximity of the external project to our sampling sites suggests a reasonable overlap in core microbial taxa, which helped validate the biological plausibility of the flooded soil data set. At the same time, differences in taxonomic composition between the two projects, driven by contrasting soil conditions and regional variations, were also expected. By examining both the overlaps and divergences, this comparison served as a critical checkpoint to ensure that the observed microbial communities reflected genuine environmental conditions.

To further assess the accuracy of the bioinformatics pipeline, the analysis was repeated on the same samples using a modified approach. In the original pipeline, reference‐based chimera removal and clustering were applied, leveraging the Silva 138 database to minimize artifacts. In the modified version, de novo methods were substituted for these steps, enabling chimera detection and OTU clustering based solely on within‐data set relationships. Taxonomic profiles generated by both pipelines were then compared. High concordance in dominant taxa across methods would indicate robustness of the pipeline, while discrepancies may be present as a result of inflated rare taxa in the de novo approach due to sequencing errors or novel chimera retention. This dual‐analysis framework helps identify systematic biases and ensures that downstream ecological interpretations are not skewed by methodological choices.

## 3 Results

### 3.1 Microbial Abundance and Composition

**Figure 2:**
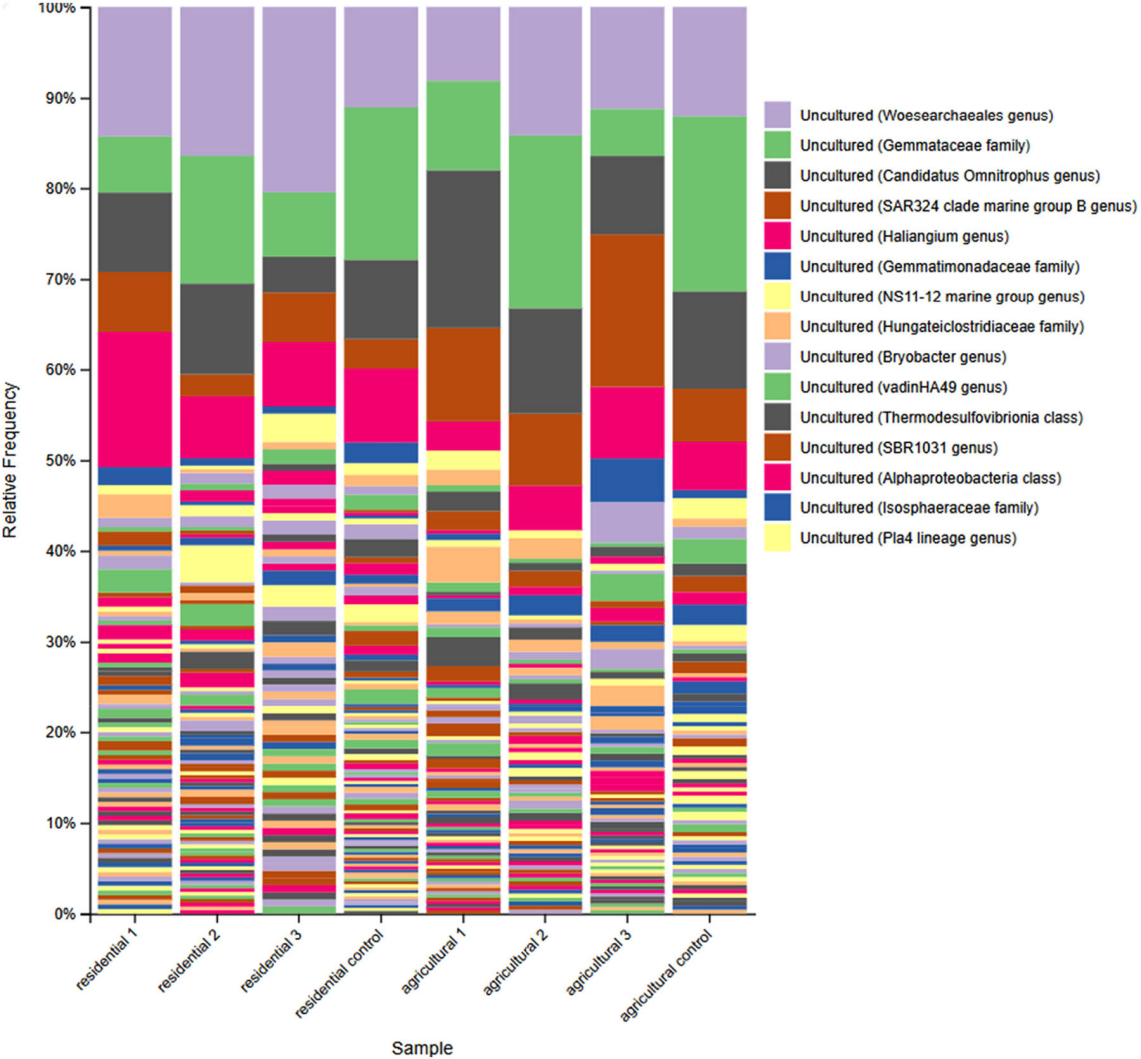
Bar plot revealing the microbial abundance for each sample. The legend table shows only the top 15 most abundant species detected. An archaeon in the *Woesearchaeales* genus was the most abundant, found in all groups. Also, found in all groups were bacteria in the *Gemmatacaeae* family, in the *Candidatus Omnitrophus* and the *Haliangium* genera, and in the SAR324 clade. None of the top 15 most abundant species were able to be classified to the species level.

**Figure 3:**
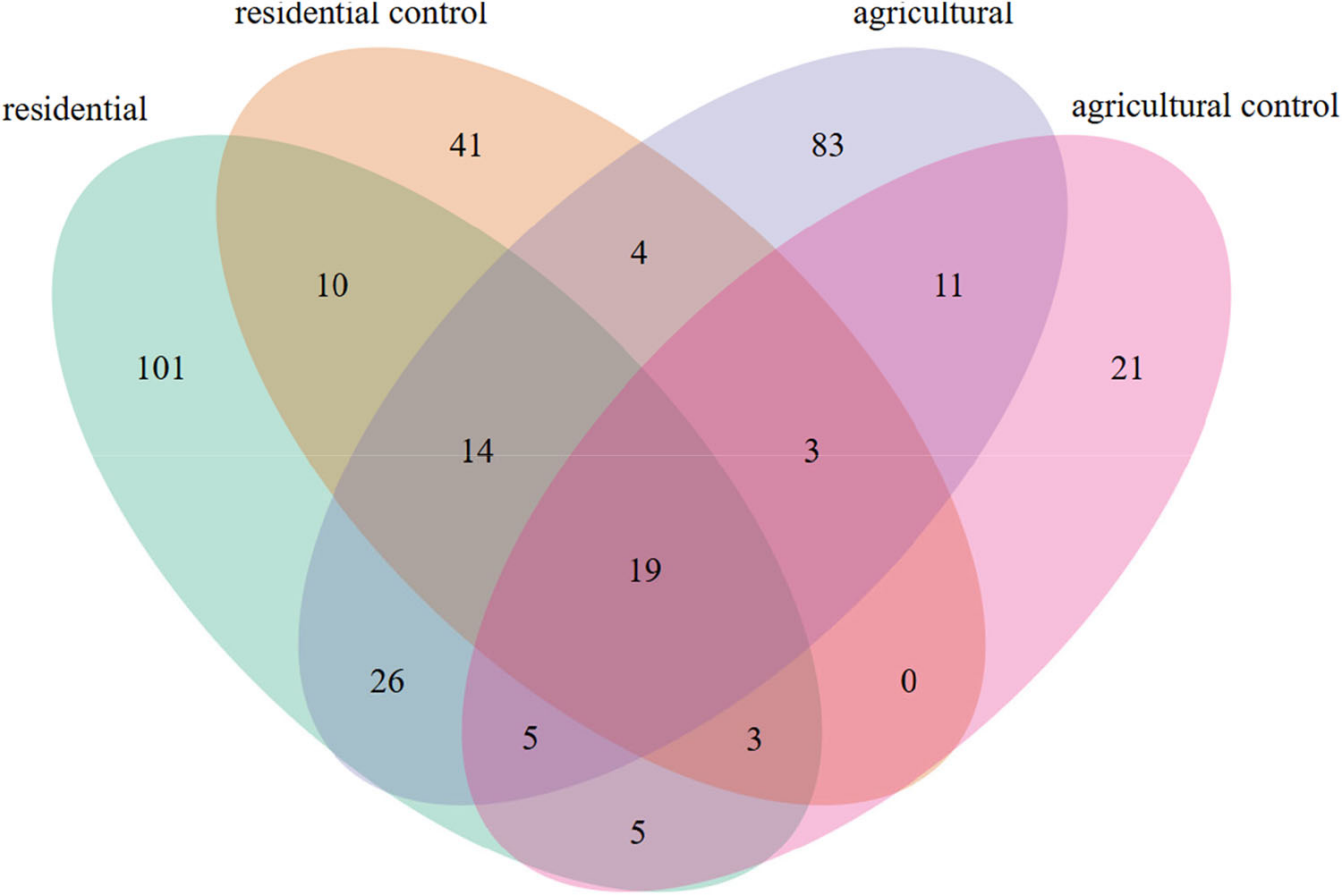
Distribution of microbial species detected among the various sample groups. Flooded groups exhibited greater microbial overlap (45 shared species) compared to non‐flooded controls (six shared species) when the common core group of 19c species was ignored. Twenty‐six taxa were exclusive to flooded environments, likely driven by water‐mediated mixing, while no species were unique to controls, underscoring floodwater's role in shaping shared communities, though sample size disparities between groups warrant caution in interpretation.

The bar plot in Figure 2 reveals the microbial abundance for each sample, with the legend table highlighting the top 15 most abundant species detected. The archaea of the order Woesearchaeales (class Nanoarchaeia) were the most abundant members of the microbial community, present in all sample groups. Bacteria from the Gemmataceae family, as well as the genera *Candidatus Omnitrophus* and *Haliangium*, were also consistently detected across all groups. Additionally, bacteria from the SAR324 (Marine group B) clade and the class vadinHA49 were found in all groups, albeit at lower abundance. Together, these taxa accounted for nearly 50% of each sample's composition. Notably, none of the top 15 most abundant species could be classified to the species level, indicating limitations in taxonomic resolution (Figure 3).

### 3.2 Differences Between Agricultural and Residential Samples

Comparative analysis revealed distinct habitat preferences among the microbial communities (Figure 2). The majority of the top abundant taxa (13 of the top 15) were more prevalent in agricultural land samples than in residential samples. The exceptions were the Woesearchaeales archaea and *Haliangium* bacteria, which were equally abundant in both environments. This divergence was further emphasized by the presence of unique taxa in each habitat: bacteria from the Moraxellaceae family and the *Candidatus Solibacter* genus were exclusively detected in land samples, whereas species including *Lysobacter sediminicola*, *Ahniella affigens*, and *Microcoleus paludosus* were unique to household samples, suggesting the establishment of habitat‐specific niches (Figure 2).

### 3.3 Microbial Overlap and Unique Taxa in Flooded and Non‐Flooded Groups

A species was considered present in a sample if its relative abundance was ≥ 0.5%, following normalization of read counts by the median sequencing depth. This threshold effectively rounds the relative abundance to the nearest integer. A core set of 19 species, including the top five most abundant taxa, was omnipresent across all conditions. Flooded groups exhibited greater microbial overlap, with 45 shared species compared to only six shared species in non‐flooded controls when the core group was excluded. Flooded residentials harbored 137 unique species absent in the non‐flooded control, including taxa associated with freshwater and soil niches, such as *Phormidium irriguum* and *Ancylothrix terrestris*, as well as species from the *Lacunisphaera* genus. Similarly, flooded agricultural samples contained 127 unique species compared to the agricultural control, including *Sulfuricurvum* sp. and members of the *Nitrospira* and *Acidibacter* genera. Notably, the *Nitrospira* genus and *Prolixibacteraceae* family were entirely absent in control samples, suggesting that flooding drives significant shifts in microbial community structure. Also remarkably, no species were exclusively shared between the control groups, whereas 26 species were uniquely shared between the flooded groups and were absent in controls. This pattern likely reflects microbial mixing due to water movement between flooded agricultural and residential areas, creating a shared ecological niche. However, the disparity in sample sizes between the flooded and control groups necessitates cautious interpretation of these findings.

### 3.4 Alpha and Beta Diversity

**Figure 4:**
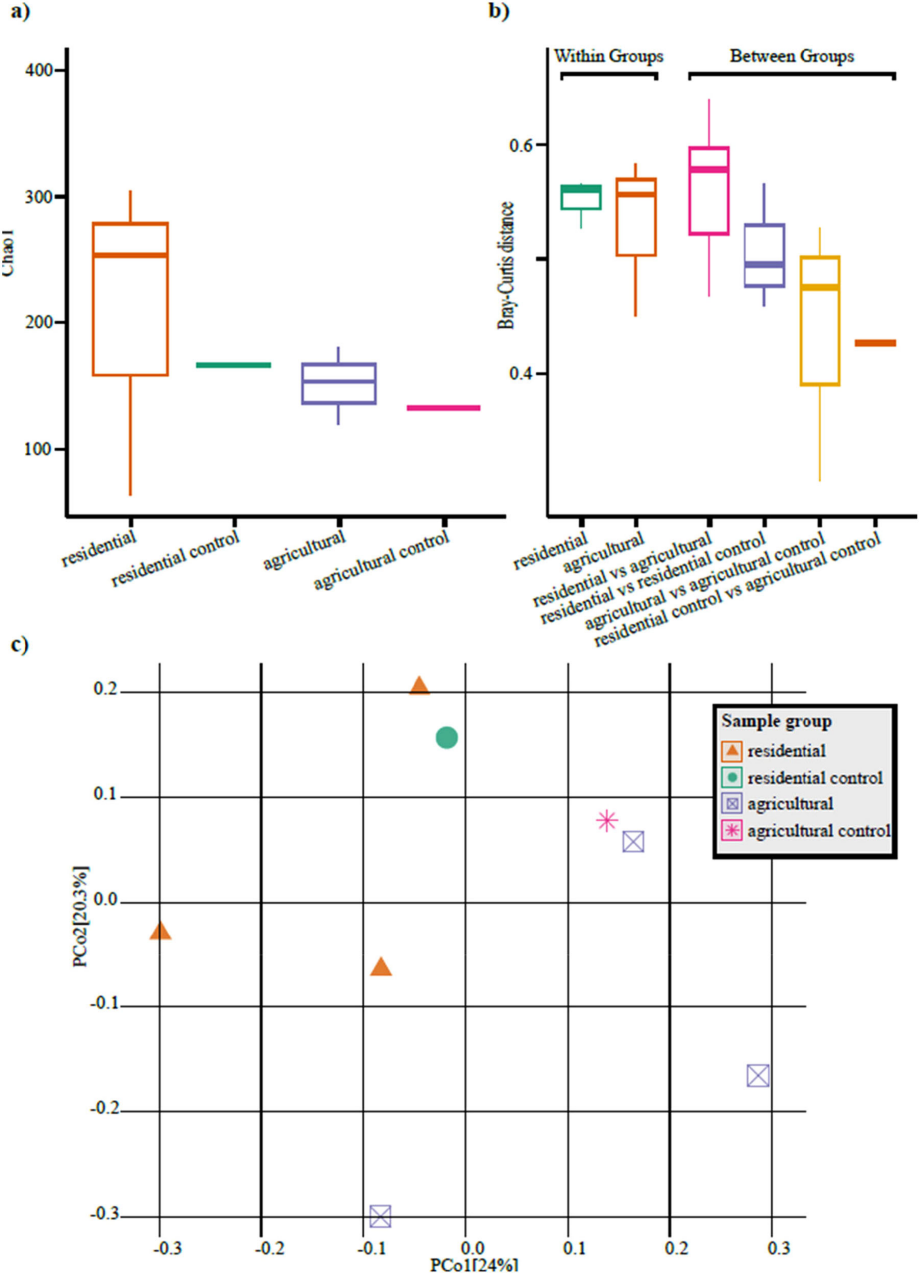
Microbial diversity analysis across sample groups. (a) Alpha diversity, measured using the Chao1 index, revealed higher species richness in flooded groups compared to non‐flooded controls, with residential samples generally exhibiting greater richness than agricultural samples. (b) Beta diversity, assessed using Bray‐Curtis dissimilarity, compared both within‐group and between‐group sample distances. While mean distances between residential and agricultural samples were comparable, agricultural samples displayed significantly higher variability. The largest mean distance was observed between residential and agricultural samples, while the smallest distance was between the two control groups. (c) Principal coordinate analysis (PCoA) based on Bray‐Curtis distances indicated a separation of sample groups into different quadrants, but the wide dispersion of sample points prevented the formation of clear clusters.

Figure 4 presents the analysis of Alpha and Beta diversities across sample groups. Figure 4a displays Alpha diversity, measured using the Chao1 index, within each group. Flooded samples exhibited higher Chao1 values compared to non‐flooded controls, indicating greater species richness and suggesting that flooding may enhance local biodiversity. Furthermore, household samples showed higher indices than land samples, implying richer microbial diversity in household environments.

Figure 4b illustrates Beta diversity, assessed using Bray‐Curtis dissimilarity. Comparisons within the same group showed a similar mean distance between household and land samples; however, land samples demonstrated significantly higher variability in pairwise distances. This may reflect greater environmental heterogeneity or more diverse ecological niches in land samples compared to households. Between samples from different groups, the largest mean Bray‐Curtis distance was between household and land samples, while the smallest distance was observed between the two control samples.

To examine statistical significance in diversity metrics, Wilcoxon Rank Sum tests were performed for both Alpha and Beta diversity. While the observed variations in species richness and community composition were not statistically significant, it is important to note that this lack of significance may be influenced by the limited statistical power of our small sample sizes rather than constituting evidence of true biological equivalence between groups.

Figure 4c depicts the results of PCoA based on Bray‐Curtis distances. While sample groups were separated into distinct quadrants, the wide dispersion of points within each group prevented the formation of tight clusters.

### 3.5 Flood‐Induced Functional Microbial Consortia in Agricultural and Residential Soils

Table 1 shows that a total of 29 microbes were identified as introduced post‐flood, categorized into 8 functional groups critical for soil recovery. Nitrogen‐fixing microbes (e.g., *Derxia sp*., *Oscillatoria sp*.) dominated residential soils, while agricultural soils hosted phosphate solubilizers (e.g., *Candidatus Koribacter*) and plant growth‐promoting rhizobacteria (e.g., *Paenibacillus chartarius*). Organic decomposers like *Geobacter sp*. (agricultural) and *Paludibaculum fermentans* (residential) demonstrated niche‐specific roles, whereas sulfur/iron cyclers (e.g., *Sulfuricurvum sp*.) and cyanobacteria (e.g., *Phormidium irriguum*) highlighted flood‐adaptive functions. Notably, *Derxia sp*. and *Candidatus Koribacter* occurred in both soils, suggesting cross‐environmental benefits. All microbes were validated through references, confirming their contributions to nutrient cycling, soil stabilization, and bioremediation in flood‐affected ecosystems.

**Table 1:** Soil‐Beneficial microbes introduced after flood by function and location.

Category	Microbe	Function/Role	Soil location	Reference
*Nitrogen Fixers*	*Derxia sp*.	Nitrogen fixation in soil and rhizospheres	Agricultural, Residential Soil	Bergey's Manual of Systematics of Archaea and Bacteria (2015); Song et al. (2017)
	*Uncultured rhizobiaceae*	Likely includes symbiotic nitrogen fixers (e.g., Rhizobium)	Residential Soil	PAMDB
	*Oscillatoria sp*.	Nitrogen‐fixing cyanobacteria in flooded soils	Residential Soil	Stal (2015); Reddy and DeLaune (2008)
	*Phormidium irriguum*	Flood‐adapted nitrogen fixation	Residential Soil	Song et al. (2017)
	*Calothrix brevissima*	Nitrogen‐fixing cyanobacteria	Residential Soil	Stal (2015)
*Phosphate Solubilizers*	*Candidatus koribacter*	Organic matter decomposition and phosphorus cycling (Acidobacteria)	Agricultural, Residential Soil	Kielak et al. (2016)
	*Uncultured acidobacterium*	Decomposer of complex organics	Agricultural Soil	Kielak et al. (2016)
	*Chitinophaga sp*.	Degrades chitin, releasing nutrients	Residential Soil	Kielak et al. (2017)
	*Geothrix sp*.	Mobilizes phosphorus via iron reduction	Residential Soil	Coates et al. (1999)
*Plant Growth‐Promoting Rhizobacteria*	*Paenibacillus chartarius*	Potential phosphate solubilization and plant growth promotion	Agricultural Soil	Grady et al. (2016)
	*Ochrobactrum sp*.	May promote plant growth via phytohormones	Agricultural Soil	Lugtenberg and Kamilova (2009)
	*Novosphingobium flavum*	Linked to the degradation of aromatic compounds and plant interactions	Agricultural Soil	NCBI Taxonomy
	*Pseudomonas sp*.	Siderophore production, phytohormones, pathogen suppression	Residential Soil	Lugtenberg and Kamilova (2009)
	*Paenibacillus contaminans*	Phosphate solubilization, plant growth promotion	Residential Soil	Grady et al. (2016)
*Organic Matter Decomposers*	*Uncultured bacteroidetes*	Degrades cellulose and other plant polymers; Key decomposers in anaerobic environments	Agricultural Soil	Fierer et al. (2007)
	*Geobacter sp*.	Breaks down organic matter via iron reduction	Agricultural Soil	Coates et al. (1999)
	*Syntrophus gentianae*	Degrades fatty acids in syntrophic associations	Agricultural Soil	McInerney et al. (2009)
	*Paludibaculum fermentans*	Breaks down polysaccharides in waterlogged soils	Residential Soil	Fierer et al. (2007)
	*Ilumatobacter fluminis*	Actinobacteria are involved in organic matter breakdown	Residential Soil	NCBI Taxonomy
	*Flavisolibacter ginsengiterrae*	Degrades plant‐derived polymers (e.g., cellulose)	Residential Soil	BRENDA Enzyme Database
*Sulfur/Iron Cyclers*	*Sulfuricurvum sp*.	Sulfur oxidation in anoxic soils	Agricultural Soil	Kodama and Watanabe (2004)
	*Uncultured delta proteobacterium*	Likely sulfate/sulfur reducers in flooded soils	Agricultural Soil	Bergey's Manual of Systematics of Archaea and Bacteria (2015)
	*Sideroxydans lithotrophicus*	Iron‐oxidizing bacterium mobilizes iron‐bound nutrients	Agricultural Soil	Emerson et al. (2013)
	*Desulfomicrobium sp*.	Sulfate reduction in flooded soils	Residential Soil	Bergey's Manual of Systematics of Archaea and Bacteria (2015)
*Cyanobacteria (Flood Roles)*	*Oscillatoriales cyanobacterium*	Stabilizes soil, fixes nitrogen, and produces organic carbon	Residential Soil	Stal (2015); Reddy and DeLaune (2008)
	*Phormidiaceae cyanobacterium*	Nitrogen fixation and organic matter production in floods	Residential Soil	Reddy and DeLaune (2008)
*Methane Cyclers*	*Uncultured methylococcales*	Methane oxidation in aerobic soils	Agricultural Soil	Knief (2015)
*Bioremediation*	*Dechloromonas agitata*	Degrades chlorinated compounds (e.g., perchlorate)	Agricultural Soil	Coates et al. (2001)
	*Georgfuchsia toluolica*	Degrades toluene and other hydrocarbons	Agricultural Soil	Kuppardt et al. (2018)

### 3.6 Pathogen Screening

Pathogen screening was conducted against the NCBI RefSeq database of well‐characterized human pathogenic species (as defined by the database's curation). This analysis revealed no canonical human pathogens in the sampled microbiota. All detected taxa were either environmental bacteria with no known human pathogenicity or lacked species‐level resolution, preventing definitive

assessment of their pathogenic potential. Crucially, this inability to resolve many taxa to the species level means we cannot confidently rule out the presence of potential pathogens, as virulence factors are often species‐ or strain‐specific. It is important to note that this screen targeted established pathogens and does not preclude the presence of opportunistic pathogens. A single exception was *Clostridium disporicum*, a Gram‐positive anaerobic bacterium rarely isolated in clinical settings, which has been linked to a rare case of postoperative infection (Plassart et al. 2012). Intriguingly, *C. disporicum* was detected exclusively in the non‐flooded agricultural control sample, suggesting either a localized environmental reservoir or potential contamination during sampling.

### 3.7 Quality Assurance

The comparison with the external aerobic soil project revealed key insights into the validity of the flooded soil samples. This project identified 346 OTUs, compared to 390 OTUs reported in the external study, with 42 OTUs (12% of this project's total) shared between the two. Notably, 8 out of the top 12 most abundant taxa in the flooded soil abundance profile were also present in the external project's data set. The substantial overlap in core taxa implies that the sampling and processing protocols were consistent and reliable, as shared environmental factors likely drove the similarity.

The comparison with the modified pipeline was used to evaluate the impact of bioinformatics methods on taxonomic resolution. The original reference‐based pipeline was compared to a modified version using de novo chimera removal and clustering, which generated 556 OTUs. All 346 OTUs from the reference‐based approach were retained in the de novo results, confirming that core taxa were consistently identified regardless of methodology. The higher OTU count in the de novo pipeline aligns with expectations, as this method detects finer‐grained diversity by clustering sequences without reliance on a reference database, capturing rare or novel lineages that reference‐based approaches might exclude.

## 4 Discussion

This study provides critical insights into the impact of flooding on microbial communities in both residential and agricultural soil environments in Chhagalnaiya, Feni. One of the most striking findings was the introduction of 29 functionally diverse microbial taxa following the 2024 Bangladesh floods, spanning eight ecological roles, including nitrogen fixers (*Derxia* sp., *Oscillatoria* sp.), phosphate solubilizers (*Candidatus Koribacter*), and sulfur cyclers (*Sulfuricurvum* sp.), which collectively enhanced nutrient cycling and soil stabilization (Fierer et al. 2007). However, as functional profiling was inferred from taxonomy and not validated metagenomically, this remains a hypothesis to be tested. In this context, “beneficial” is defined by their documented role in supporting soil health and ecosystem resilience through key biogeochemical processes, ultimately creating conditions that support crop productivity. Nitrogen‐fixing cyanobacteria dominated residential soils, while agricultural soils specialized in sulfur oxidation, reflecting niche‐specific adaptations to flood conditions. Notably, pathogens were largely absent, with *Clostridium disporicum* detected exclusively in a non‐flooded control, contrasting reports of flood‐associated pathogen proliferation (Ahmed et al. 2021). These findings underscore flooding's dual role as both an ecological disruptor and a catalyst for microbial resilience.

The dual presence of *Derxia sp*. in agricultural and residential soils suggests its ecological plasticity, enabling cross‐environmental nutrient exchange critical for post‐flood recovery (Brammer 2014). Residential soils uniquely hosted *Lysobacter sediminicola*, a hydrocarbon‐degrading bacterium, while agricultural soils retained *Geobacter sp*., which facilitates iron‐mediated organic decomposition (Coates et al. 1999). Despite 16S rRNA sequencing's limited resolution for uncultured taxa like *Candidatus Koribacter* (Quast et al. 2012), cross‐validation with external datasets (NCBI BioProject PRJNA1123412) confirmed core taxa reliability. Flooding increased microbial diversity, with flooded groups sharing 44 species versus 6 in non‐flooded controls, suggesting floodwaters drive microbial dispersal. Dominant taxa, including *Woesearchaeales* archaea and *Gemmataceae* bacteria, comprised ~50% of communities. Habitat‐specific niches emerged: *Moraxellaceae* and *Candidatus Solibacter* were agricultural‐exclusive, while *Lysobacter sediminicola* and *Microcoleus paludosus* were residential‐specific. Challenges included unresolved species‐level classification of abundant taxa and the detection of *Clostridium disporicum* solely in non‐flooded controls, complicating origin interpretation. Alpha diversity (Chao1) was higher in flooded and residential samples, while Beta diversity indicated land variability, though without statistical significance (Wilcoxon test). While visual trends in the PCoA suggest potential flood‐induced shifts, the analysis does not provide statistically robust evidence that flooding significantly altered the microbial community structure in this data set.

Our findings align with global studies demonstrating that flooding enhances microbial nutrient cycling (Fierer et al. 2013) and reflect Bangladesh's traditional floodplain agriculture, where microbes drive silt deposition and soil fertility (Islam et al. 2021). However, the absence of waterborne pathogens contrasts with reports of flood‐associated outbreaks (Ahmed et al. 2021). While early‐stage sampling or methodological differences (e.g., metagenomics vs. culture‐based detection) may contribute, local environmental conditions could also play a key inhibitory role. For instance, the slightly alkaline pH (7.8–8.2) and elevated organic matter content observed in our soil samples may have created an environment less favorable for the proliferation of common enteric pathogens, which often thrive in more neutral conditions. Furthermore, potential biotic competition from the established, resilient indigenous microbiota (e.g., Woesearchaeales, Gemmataceae) could have outcompeted incoming allochthonous pathogenic species, preventing their successful colonization. This highlights the need to consider local edaphic factors when assessing the public health risks of flooding. The dominance of *Woesearchaeales* archaea and *Gemmataceae* bacteria, previously observed in temperate floodplains (Liu et al. 2018; Wiegand et al. 2018), extends their ecological roles to tropical deltaic ecosystems, underscoring their universal significance. These insights offer actionable strategies: bioaugmentation with *Paenibacillus spp*. could improve phosphorus availability for crops (Grady et al. 2016), while *Lysobacter sediminicola* may mitigate urban waterlogging impacts. Integrating microbial insights into Bangladesh's climate adaptation frameworks could align flood management with ecological recovery. Future research should prioritize multi‐omics approaches to resolve metabolic pathways and longitudinal studies to track microbial succession. By harnessing flood‐induced microbial communities, we transform vulnerability into resilience, redefining flood mitigation in deltaic regions. Our findings also align with studies showing that flooding reshapes microbial communities by facilitating dispersal and creating new ecological niches (Fierer et al. 2013). The increased microbial overlap in flooded environments supports the hypothesis that water movement promotes microbial mixing (Baldwin et al. 2020). However, the absence of canonical human pathogens contrasts with reports of pathogen proliferation in floodwaters (Ahmed et al. 2021; Islam et al. 2021), potentially due to differences in sampling locations, timing, or methodological approaches. Our use of metagenomics, which offers a more comprehensive view of microbial diversity than traditional culture‐based methods, reinforces the importance of advanced sequencing technologies for accurate pathogen detection and monitoring (Quince et al. 2017; Gilbert et al. 2014).

Key limitations include taxonomic resolution challenges due to 16S rRNA sequencing's inability to resolve species‐ or strain‐level functions (Bokulich et al. 2018), a small sample size reducing statistical power (McMurdie and Holmes 2013), and reliance on incomplete pathogen databases (NCBI Pathogen Database 2024). A primary limitation of this study is the reliance on 16S rRNA gene amplicon sequencing to infer microbial function. As functional traits are not always conserved at the taxonomic levels resolved here, these predictions remain speculative. Future studies employing shotgun metagenomics are essential to directly characterize the metabolic potential and functional genes of the microbial community. Another limitation was the lack of technical replication for non‐flooded control samples, a consequence of field access constraints. While these controls provide a valuable preliminary baseline, comparisons involving them should be interpreted with caution. Future studies with larger sample sizes are needed to confirm the robustness of these initial findings. The SILVA 138 reference database, while cost‐effective for broad community profiling, lacks rigorous species‐level curation, often limiting classifications to genus or family ranks, particularly for uncultured taxa. This granularity gap complicates pathogen screening and functional annotation. Additionally, the study's cross‐sectional design precludes causal inferences about flood‐induced microbial shifts. Future studies should integrate shotgun metagenomics to resolve strain‐level roles and longitudinal sampling to track microbial succession, addressing these constraints and enhancing ecological insights.

These findings highlight the profound impact of flooding on microbial communities and underscore the need for robust monitoring systems to assess public health risks in flood‐prone regions. The identification of unique microbial taxa in flooded environments provides a foundation for further research into their ecological roles and potential applications, such as bioremediation or agricultural improvement. Future studies should prioritize longitudinal sampling to track microbial dynamics, expand sample sizes to improve statistical power, and integrate functional metagenomics to elucidate the metabolic capabilities of flood‐associated microbes. Additionally, shotgun metagenomics could enhance taxonomic resolution and detect virulence or antibiotic resistance genes, offering a more comprehensive view of public health risks. Developing standardized protocols for pathogen screening and fostering collaboration between environmental scientists and public health agencies will be critical for translating these insights into actionable strategies. By adopting these approaches, we can better prepare for and mitigate the health and environmental impacts of flooding in vulnerable regions like Bangladesh.

## Author Contributions

M.R. and M.J. developed the protocol, methodology, conceived and coordinated the study, and reviewed the manuscript. TR and MJ interpreted laboratory data, cleaned and finalized the data set, performed the descriptive analyses, and prepared the first draft of the manuscript. S.M.A.H. and F.T.Z.K. were involved in the laboratory work and analysis of laboratory data and provided intellectual input to the manuscript. M.S.I., Z.H.M., and M.M.K. critically reviewed the manuscript and provided intellectual input. All authors reviewed subsequent drafts of the manuscript and approved the final version. All authors had full access to all the data in the study and accepted the responsibility for the integrity of the data, accuracy of the data analysis, and publication.

## Ethics Statement

This environmental study involved soil sample collection and analysis, with no human or animal subjects. Ethical approval was not required. Sampling followed local regulations, and permissions were obtained from landowners and authorities.

## Conflicts of Interest

The authors declare no conflcits of interest.

## Transparency Statement

The corresponding author affirms that this manuscript is an honest, accurate, and transparent account of the study being reported; that no important aspects of the study have been omitted, and that any discrepancies from the study as planned (and, if relevant, registered) have been explained.

## Data Availability

All the required sequence data are available on request.
